# Drug-Induced vs. Viral Maculopapular Exanthem—Resolving the Dilemma

**DOI:** 10.3390/dermatopathology9020021

**Published:** 2022-05-07

**Authors:** Sujay Khandpur, Rhea Ahuja

**Affiliations:** Department of Dermatology and Venereology, All India Institute of Medical Sciences, New Delhi 110029, India; ahujarhea1@gmail.com

**Keywords:** maculopapular, exanthema, drug, viral

## Abstract

Maculopapular exanthem is a commonly encountered presentation in routine clinical practice, and differentiation between its two most common etiologies, i.e., viral- and drug-induced, often poses a diagnostic dilemma. Clinical, hematological and biochemical investigations are seldom reliable in distinguishing between a drug reaction and a viral exanthem. Certain key histopathological features such as the presence of a moderate degree of spongiosis, extensive basal cell damage with multiple necrotic keratinocytes and dermal infiltrate rich in eosinophils or lymphocytes and histiocytes may favor a drug exanthem, while distinctive epidermal cytopathic changes and lymphocytic vasculitis point towards a viral etiology. Similarly, notable immunohistochemical markers such as IL-5, eotaxin and FAS ligand may support a diagnosis of a drug-induced maculopapular eruption. Histopathological and immunohistochemical evaluations may help in distinguishing between the two etiologies when faced with a clinical overlap, especially in patients on multiple essential drugs when drug withdrawal and rechallenge is not feasible.

## 1. Introduction

Maculopapular exanthem is characterized by an acute and generalized eruption of erythematous macules and papules without overlying scaling. It is a common presentation in day-to-day clinical practice, and differentiating between its two most common etiologies, i.e., viral- and drug-induced, is often perplexing. An eruption occurring after drugs having been initiated for treatment of an underlying infection further adds to the diagnostic dilemma. Establishing an etiological diagnosis aids the clinician in deciding whether to interrupt a culprit drug or to continue a wrongly incriminated and possibly life-saving drug. This review aims to distinguish between viral- and drug-induced exanthem, with a special focus on histopathological features.

## 2. Clinical Features

**Figure 1:**
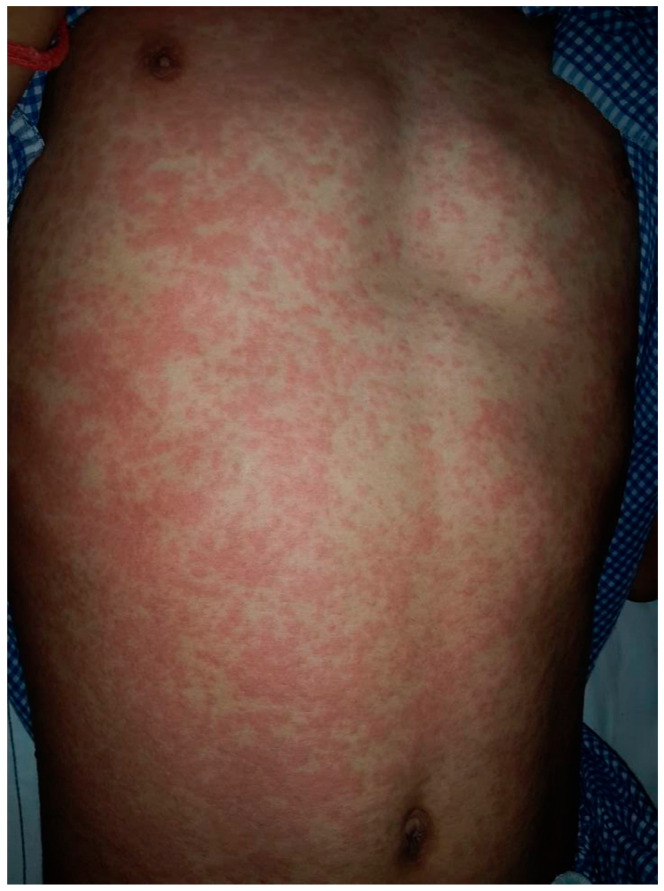
Maculopapular drug exanthem on abdomen.

Maculopapular eruption is the most common presentation, accounting for around 90% of cases among 1317 patients with cutaneous drug hypersensitivity reactions [1]. The commonly implicated drugs include antibiotics such as beta-lactams and suphonamides, anti-convulsants and non-steroidal anti-inflammatory drugs [2]. The cutaneous eruption usually occurs within 7 to 10 days (range 5 to 21 days) after treatment, starting from the trunk and proximal extremities. Distinctive features of a drug-induced exanthem include the presence of pruritus, confluence of the rash on dependent areas, facial involvement and purpuric lesions on lower extremities [3]. A temporal correlation with drug intake is important to ascertain causation, and in a previous study re-appearance of the rash on drug provocation was an essential diagnostic criterion for defining definite drug-induced exanthem [4] [Figure 1].

**Figure 2:**
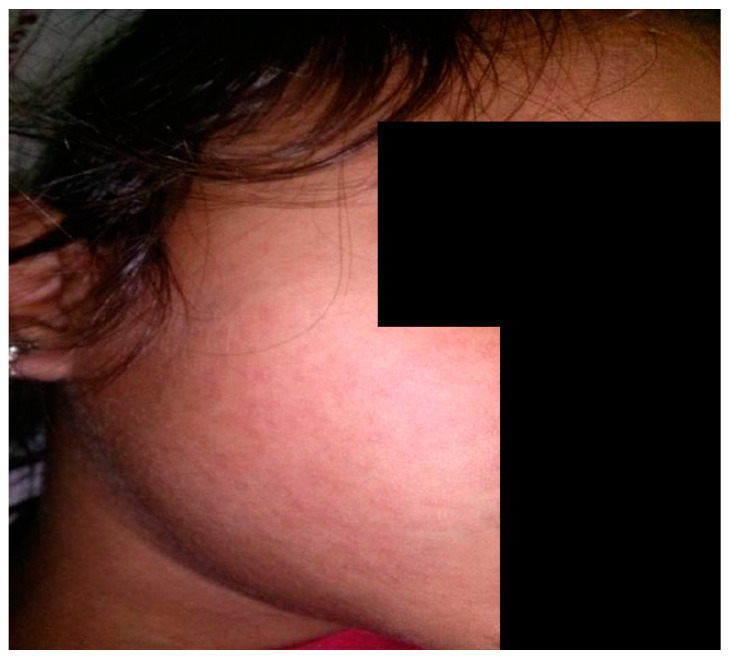
Viral exanthem on face of child.

Viral exanthem, on the other hand, presents with prodromal symptoms such as fever, conjunctivitis and rhinorrhea, followed by the appearance of usually non-pruritic erythematous macules and papules in a patterned distribution with a cephalocaudal spread [3]. Distinctive mucosal involvement or enanthems in the form of Koplik spots (measles), Forchheimer spots (rubella) and Nagayama spots (roseola infantum) can also be noted. Interestingly, certain exanthems occur after drug initiation in a previously infected patient, such as the generalized maculopapular eruption occurring after ampicillin intake in a patient with infectious mononucleosis [5] [Figure 2].

## 3. Hematological and Biochemical Investigations

Blood eosinophilia may support a diagnosis of drug-induced exanthem. Singh et al. found the median absolute eosinophil count to be higher than normal in patients with drug exanthem, whereas they were within normal limits in those with viral exanthem, although the difference was not statistically significant [4]. Yawalaker et al. have also described that cutaneous drug eruptions may be associated with blood and tissue eosinophilia [6]. Although blood eosinophilia is not pathognomonic for maculopapular drug exanthem, the degree of eosinophilia usually correlates with the severity of drug reactions, more often in drug reactions with eosinophilia and systemic symptoms (DRESS) [7]. Biochemical investigations such as hepatic and renal function panels are usually normal, unless the patient is suffering from DRESS syndrome.

C-reactive protein levels also do not play a significant role in establishing an etiological diagnosis of maculopapular eruption. Although the levels are usually higher in drug exanthem compared to viral exanthem, the difference is not statistically significant [4,8].

## 4. Histopathology

Lever’s Textbook of Dermatology and Mckee’s Pathology of Skin have emphasized that the histological findings of an exanthematous drug eruption are often indistinguishable from those of viral exanthems, although the presence of eosinophils may favor a drug reaction [9,10]. Ackerman also believed that “drugs can elicit any of the nine basic patterns of inflammatory diseases in the skin, and none of those patterns is specific for a drug eruption” [11]. Nonetheless, various studies have highlighted certain histopathological pointers for both drug- and viral-induced exanthema, albeit they may not be pathognomonic.

Seitz et al. in their study on 91 patients of drug-induced exanthema described the most common reaction pattern to be the combined type of spongiotic and interface pattern, followed by the perivascular (15/26), spongiotic (10/13), vacuolar interface (6/10) and lichenoid interface patterns (4/5). Most importantly, no specific reaction pattern could be significantly attributed to drug-induced or non-drug-induced exanthem. Parakeratosis was present in a slightly but not significantly higher proportion of non-drug-induced exanthem than drug-induced exanthem. No significant differences in occurrence of dyskeratotic keratinocytes, extent of lymphocyte exocytosis or density of the dermal lymphocytic infiltrate were observed. Moreover, there was no significant difference in the presence of papillary dermal edema, accompanying vasculitis or extravasation of erythrocytes. A comparison of drug-induced exanthema and non-drug-induced exanthem did not yield a significant difference in the number of eosinophils within the dermal infiltrate. The results regarding the sensitivity (62.9%), specificity (41.1%) and positive (40.7%) and negative predictive values (69.7%) of a correct diagnosis showed that no valid conclusions can be drawn from histologic evaluations of skin biopsy specimens [12].

**Figure 3:**
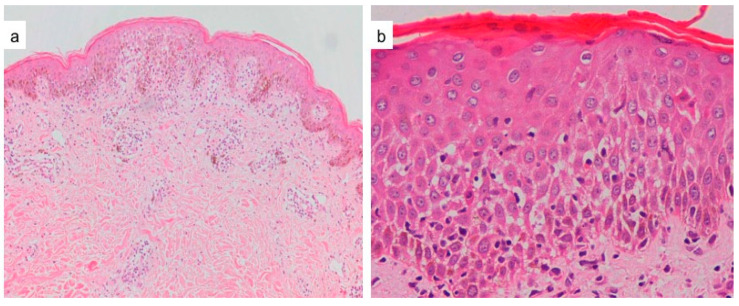
(**a**) Drug exanthem to tranexamic acid, showing spongiosis, focal vacuolar interface changes with papillary dermal perivascular and interstitial infiltration of lympho-histiocytes and eosinophils (H&E ×40). (**b**) Drug exanthem to rifampicin, showing parakeratosis, spongiosis, necrotic keratinocytes in upper layers of the epidermis, lymphocytic exocytosis and papillary dermal infiltration of lymphocytes and eosinophils (H&E ×400).

**Figure 4:**
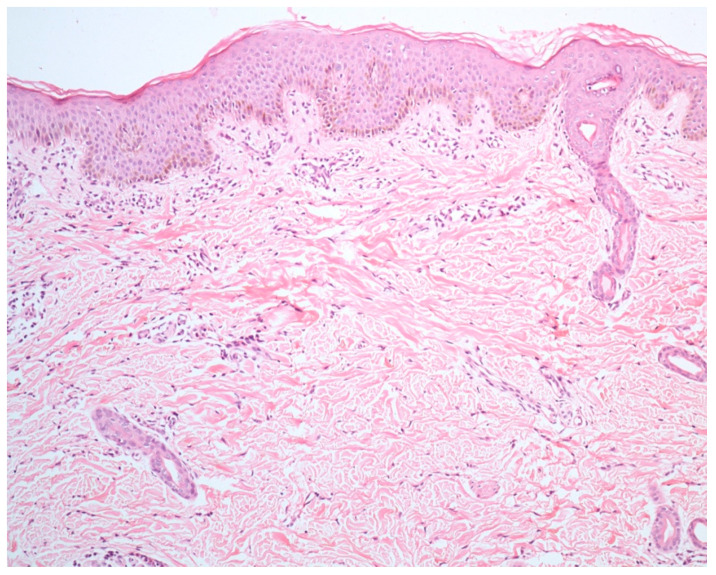
Viral exanthem showing normal epidermis with sparse perivascular infiltration of lymphocytes and occasional eosinophils (H&E ×40).

However, other authors have noted certain distinctive findings that may point towards either a drug-induced or a viral exanthem [Figure 3 and Figure 4]. We will now highlight the important differences proceeding from the epidermis to the dermis.

### 4.1. Spongiosis

Naim et al. while evaluating 60 biopsy specimens noted that spongiosis was the most consistent feature (97%) in maculopapular drug eruptions, and it was mostly focal [13]. Other authors also corroborated this finding, with spongiosis being noted in significantly higher number of drug eruption cases (50%) compared to viral exanthem (17%), however it was of a moderate-severe degree [4]. Spongiosis may be associated with inflammatory cell exocytosis variably noted in 38–100% cases [13,14]. Usually eosinophilic or lymphocytic exocytosis is noted, but invasion by solitary neutrophils is an important clue towards a drug etiology [13]. The presence of follicular spongiosis is uncommon, present in up to 8% of exanthems of either etiology and cannot differentiate between the two [4,13].

### 4.2. Viral Cytopathic Changes

Some viral exanthems can be recognized by their distinctive features, such as ballooning and multinucleated keratinocytes in measles and keratinocytes with shrunken nuclei with margination of the nucleoplasm in infections by herpesviruses, although these changes are relatively rare. Liersch et al. noted certain distinct histopathological features in measles characterized by multinucleated keratinocytes and individual and grouped necrotic keratinocytes in the epidermis with pronounced folliculo-sebaceous and acrosyringeal involvement [15].

### 4.3. Necrotic Keratinocytes and Basal Cell Damage

This finding is also noted more often in drug-induced (21% and 29%) than in viral exanthem (4% and 8%). The presence of many necrotic keratinocytes and severe basal cell damage is an important clue towards a drug’s etiology [4]. Weyer et al. have shown that the most common histopathological pattern of maculopapular drug eruption is vacuolar interface dermatitis, noted in 97% of cases [16]. The degree of interface change is highly variable, ranging from slight vacuolar damage with few necrotic keratinocytes to severe vacuolar alterations with many necrotic keratinocytes, even at higher levels in the epidermis. However, severe interface changes are more commonly observed in a severe cutaneous adverse drug reaction such as erythema multiforme, Stevens–Johnson syndrome and toxic epidermal necrolysis rather than maculopapular drug eruption.

### 4.4. Chronic Dermal Inflammatory Infiltrate

A lymphocytic and histiocytic dermal infiltrate was observed in a significantly higher proportion of drug exanthema (62.5%) compared to viral exanthema (12.5%) biopsies [4]. The infiltrate may be perivascular, interstitial or associated with necrotic keratinocytes at the dermo-epidermal junction. The lymphocytes may be atypical and large, as noted in 16.7–35% of biopsy samples [4,14].

### 4.5. Eosinophilic Dermal Infiltrate

This finding is noticed in a significantly higher proportion of drug exanthem (52%) compared to viral exanthem (12.5%) biopsies [4]. Weyer et al. in their study on maculopapular eruption found that besides eosinophils, neutrophils are also an important constituent of the dermal infiltrate. Although eosinophils are more common, because they are seen in such a wide variety of diseases, they are less distinctive for drug eruptions. An infiltrate of neutrophils is rarer but of greater diagnostic importance. Sparse perivascular and interstitial infiltration of neutrophils and eosinophils with subtle vacuolar changes at the dermoepidermal junction is virtually diagnostic of a drug eruption [16].

### 4.6. Lymphocytic Vasculitis

Viral exanthem may be associated with lymphocytic vasculitis, a finding which is uncommon in drug exanthema [16]. A dense perivascular cuff of lymphocytes with RBC extravasation has also been observed in COVID-associated maculopapular eruption, although more commonly in a livedo-like or erythema-multiforme-like clinical presentation [17].

Other discernible features such as hyperkeratosis, parakeratosis, fibrin globules in the stratum corneum, acanthosis, colloid bodies in the papillary dermis, congested capillaries and pigment incontinence may be observed more commonly in biopsies from maculopapular drug eruption compared to viral exanthem, although these alone may not be sufficient in differentiating the two [4].

## 5. Immunohistochemical Techniques

Attempts have been made to histologically diagnose drug-induced exanthem and differentiate it from viral exanthem using immunohistochemical techniques. Using CD4, CD8, FAS ligand, IL-5 and eotaxin markers, differentiation between the two entities have been made. FAS–ligand in the serum and tissue is elevated in most cases of maculopapular drug exanthem, but it is normal in viral exanthem [18,19].

IL-5 and eotaxin cause activation and recruitment of eosinophils, thereby contributing to the development of skin inflammation in drug-induced maculopapular exanthems. Yawalkar et al. performed an IHC analysis of the dermal mononuclear infiltrate in drug exanthem biopsies and compared it with normal skin in control subjects. IL-5 expression was mainly found among mononuclear cells of the inflammatory infiltrate. Immunoreactivity for eotaxin, RANTES (regulated on activation, normal T cell expressed and secreted), IL-8 and to a lesser extent monocyte chemotactic protein-3 were also seen in the mononuclear cells. In addition, resident cells (i.e., endothelial cells and keratinocytes) also demonstrated positivity for eotaxin, RANTES and IL-8 [6].

The chronic dermal inflammatory infiltrate in a drug exanthem is primarily composed of CD3+ T cells. Within T-lymphocytes, CD4+ T cells are noted predominantly in the perivascular location, whereas equal proportions of CD4+ and CD8+ T cells are located at the dermoepidermal junction and in the epidermis [20]. Up to 20% of the infiltrating T cells in drug-induced maculopapular exanthem express perforin and granzyme B, which are important components of cell-mediated cytotoxic reaction. In addition to T cells, eosinophils are also present within the dermal infiltrate. These may also contribute to the generation of tissue damage via the release of various toxic granule proteins, such as eosinophilic cationic protein, major basic protein and eosinophil peroxidase.

Bellini et al. performed an IHC evaluation of the cutaneous cytokine expression in maculopapular eruptions (36 patients with drug exanthem, 30 patients with viral or bacterial exanthem). They also noted a higher number of IL-5-, perforin- and granzyme-B-positive cells in drug-induced exanthem. The distribution of cytokine positivity gradually decreased with an increase in the duration between the onset of the exanthem and timing of the biopsy. In contrast, FAS-L, IL-10 and INF-γ were expressed in biopsies from both groups (~40%), so their levels were non-discriminatory. However, FAS-L levels were disproportionately raised (~90%) in those patients with amoxicillin-induced exanthem, so it may represent a differentiating tool only for an amoxicillin-induced rash [21].

In addition to drug exanthem, IHC may help confirm a suspicion of viral exanthem, e.g., the staining of skin biopsies with anti-measles virus (MeV) nucleoprotein and anti-MeV phosphoprotein can be of great value in confirming the diagnosis of this exanthem [15].

## 6. Conclusions

Maculopapular exanthem is a commonly encountered presentation in routine clinical practice, and differentiation between its two most common causes, i.e., viral- and drug- induced, may be perplexing. Clinical features are often overlapping, although temporal correlations with drug intake and drug rechallenge may point towards a drug-induced maculopapular eruption. Besides blood eosinophilia, other hematological and biochemical investigations are often unreliable in distinguishing between the two etiologies. Certain key histopathological features such as the presence of a moderate degree of spongiosis, extensive basal cell damage with multiple necrotic keratinocytes and dermal infiltrate rich in eosinophils or lymphocytes and histiocytes may favor a drug exanthem, while distinctive epidermal cytopathic changes and lymphocytic vasculitis point towards a viral etiology. Similarly, some immunohistochemical markers such as IL-5, eotaxin and FAS ligand may support a diagnosis of a drug-induced maculopapular eruption. The important differences between drug-induced and viral induced maculoapapular exanthem are highlighted in Table 1.

## Figures and Tables

**Table 1:** Differentiation between drug-induced and viral maculopapular exanthem.

Maculopapular Eruption	Drug-Induced	Viral-Induced
**Clinical features**		
Prodromal features	Usually absent	Fever, conjunctivitis, rhinorrhea, myalgia, arthralgia
2. Rash	Pruritic, may be confluent on dependant areas ± facial involvement	Usually non-pruritic, with patterned distribution ± enanthems
3. Distribution	Haphazard distribution. Starts from trunk and proximal extremities	Cephalocaudal spread
4. Temporal co-relation	Within 7–10 days after drug intake, improves on withdrawal, reappears on re-challenge	None
**Hematological investigations**		
Blood eosinophilia	Higher median absolute eosinophil count	Lower median absolute eosinophil count
**Histopathology**		
Spongiosis	More common(50%), usually moderate-severe degree	Less common(16.8%), usually mild
2. Cytopathic changes	None	Seen in some infections eg. ballooning and multinucleated keratinocytes in measles, keratinocytes with shrunken nuclei in infection by herpesviruses
3. Necrotic keratinocytes and basal cell damage	More common (20.8% and 29%) Many necrotic keratinocytes and basal cell damage clue to a drug exanthem	Less common (4% and 8.3%)
4. Chronic dermal inflammatory infiltrate	More common (54–91.3%)	Less common (12.5%)
5. Eosinophilic dermal infiltrate	More common (45–62.5%)	Less common (12.5–20%)
6. Neutrophilic exocytosis/neutrophils in dermal infiltrate	More common	Less common
**Immunohistochemical evaluation**		
Serum IL-5 and eotaxin	Higher levels, role in recruiting eosinophils	Lower expression
2. Perforin and granzyme-B	Higher levels, expressed by infiltrating cytotoxic T cells	Lower levels
3. Serum FAS ligand	Levels disproportionately higher in patients with amoxicillin- induced drug eruption	Levels may be raised
